# Development and Pilot Implementation of TACTICS VR: A Virtual Reality-Based Stroke Management Workflow Training Application and Training Framework

**DOI:** 10.3389/fneur.2021.665808

**Published:** 2021-11-11

**Authors:** Rebecca J. Hood, Steven Maltby, Angela Keynes, Murielle G. Kluge, Eugene Nalivaiko, Annika Ryan, Martine Cox, Mark W. Parsons, Christine L. Paul, Carlos Garcia-Esperon, Neil J. Spratt, Christopher R. Levi, Frederick R. Walker

**Affiliations:** ^1^Centre for Advanced Training Systems, The University of Newcastle, Callaghan, NSW, Australia; ^2^School of Biomedical Sciences and Pharmacy, College of Health Medicine and Wellbeing, The University of Newcastle, Callaghan, NSW, Australia; ^3^Hunter Medical Research Institute, New Lambton Heights, NSW, Australia; ^4^School of Medicine and Public Health, College of Health Medicine and Wellbeing, The University of Newcastle, Callaghan, NSW, Australia; ^5^Department of Medicine and Neurology, Melbourne Brain Centre, Royal Melbourne Hospital, Melbourne, VIC, Australia; ^6^Department of Neurology, John Hunter Hospital, New Lambton Heights, NSW, Australia; ^7^The Sydney Partnership for Health, Education, Research and Enterprise (SPHERE), Sydney, NSW, Australia

**Keywords:** virtual reality, technology, medical education, medical training, stroke workflow, hyper-acute stroke management

## Abstract

Delays in acute stroke treatment contribute to severe and negative impacts for patients and significant healthcare costs. Variability in clinical care is a contributor to delayed treatment, particularly in rural, regional and remote (RRR) areas. Targeted approaches to improve stroke workflow processes improve outcomes, but numerous challenges exist particularly in RRR settings. Virtual reality (VR) applications can provide immersive and engaging training and overcome some existing training barriers. We recently initiated the TACTICS trial, which is assessing a “package intervention” to support advanced CT imaging and streamlined stroke workflow training. As part of the educational component of the intervention we developed TACTICS VR, a novel VR-based training application to upskill healthcare professionals in optimal stroke workflow processes. In the current manuscript, we describe development of the TACTICS VR platform which includes the VR-based training application, a user-facing website and an automated back-end data analytics portal. TACTICS VR was developed *via* an extensive and structured scoping and consultation process, to ensure content was evidence-based, represented best-practice and is tailored for the target audience. Further, we report on pilot implementation in 7 Australian hospitals to assess the feasibility of workplace-based VR training. A total of 104 healthcare professionals completed TACTICS VR training. Users indicated a high level of usability, acceptability and utility of TACTICS VR, including aspects of hardware, software design, educational content, training feedback and implementation strategy. Further, users self-reported increased confidence in their ability to make improvements in stroke management after TACTICS VR training (post-training mean ± SD = 4.1 ± 0.6; pre-training = 3.6 ± 0.9; 1 = strongly disagree, 5 = strongly agree). Very few technical issues were identified, supporting the feasibility of this training approach. Thus, we propose that TACTICS VR is a fit-for-purpose, evidence-based training application for stroke workflow optimisation that can be readily deployed on-site in a clinical setting.

## Introduction

Delays in time-to-reperfusion treatment for stroke patients can have severe and negative impacts on outcomes (1). Even relatively short delays can have enduring effects, resulting in tissue damage and an estimated loss of 1.9 million brain cells per minute of severe brain ischemia (2). Patient impairments, resulting from treatment delay, also place a significant burden on the community, with each hour of delay resulting in loss of 0.77 quality-adjusted life years and >$10,000 AUD in associated costs (3, 4). Given the consequences associated with reperfusion treatment delay, efficient and accurate treatment particularly in workflow processes (which is dependent upon healthcare staff training) is critical to ensuring optimal patient outcomes.

Variability in clinical care is known to be a major contributor to the delayed delivery of treatments and in turn sub-optimal patient outcomes (5), particularly in rural, regional and remote (RRR) areas. Of note, “door-to-reperfusion treatment delivery” time in rural NSW Australia hospitals is >100 min (6), whereas guidelines target <60 min (and ideally <45 min) (7). Similarly, transfers from rural hospitals to comprehensive specialized stroke centers for endovascular clot retrieval have median “door-in-door-out” times of 214 min (8). In a 2019 Australian National Stroke Audit, only 52% of stroke patients in Australia were assessed using a validated stroke screen upon arrival in the emergency department, while only around 10% of stroke patients received intravenous thrombolysis (IVT) treatment and 32% of patients were treated within 60 min of arrival at the hospital (9). Thus, improvements are needed to align stroke management workflow with existing guidelines and deliver optimal patient outcomes.

There are numerous challenges in delivering best-practice stroke management, particularly in RRR settings in Australia (10) and worldwide (11). One significant contributor to delays at RRR sites is the nature of the workforce, which is typically more junior, non-specialist and transient compared to metropolitan hospitals, as well as having less access to continuing professional development training (12–14). While the transitory and less-specialized nature of the RRR workforce is structural and difficult to modify, knowledge of best-practice and the confidence to apply it are in-principle tractable. However, many healthcare professionals involved in hyper-acute stroke management indicated they have received limited practical or “case-based” procedural training (15). Thus, novel training approaches are required.

Delivering stroke workflow optimisation training (particularly at RRR sites) is challenging. Training is typically delivered by stroke specialists *via* traditional face-to-face workshops, which are occasional, have limited reach and are logistically complex. Specialists that can provide high-quality training have limited capacity to travel on a regular basis. Constraints of this approach have been exacerbated by the current Covid-19 pandemic (16). Training typically centers around case studies and simulation-based learning and delivery is costly, slow, difficult to deploy and often not scalable. Knowledge transfer typically defaults to passive paper- or PowerPoint-based approaches. The outcome is limited trainee engagement, modest consumption of training and limited knowledge transfer. While online-based teaching approaches can be interactive, effective and scalable, their effectiveness in skill development, decision-making and workflow optimisation training is less studied.

Implementation trials assessing targeted approaches to improve stroke workflow processes have produced some improvements in clinical care and patient outcomes. For example, embedding dedicated stroke coordinators into rural NSW hospitals increased patient access to stroke units, the use of care plans and allied health assessments (17). As a result, patients were 89% more likely to be discharged home (17). Further, implementation of a pre-hospital stroke assessment tool for ambulance officers and pre-hospital notification system effectively increased IVT treatment rates (from 4.7 to 21.4%) (18). Despite some improvements in stroke outcomes, trials have highlighted the passive nature of existing training approaches and highlighted a need for an integrated approach to improve workflow training (e.g., by integrating practical and interactive training elements) (19, 20). Interventions conducted in a research setting highlight the significant resource costs of implementing new training strategies and the need to consider long-term sustainment in real-world settings to achieve long-term gains. Our work developing and testing implementation strategies for optimal stroke care support the need for novel tools and resources that effectively support both training uptake and engagement (15, 19, 21, 22). It is now critical to assess the feasibility of implementing novel approaches within the clinical workplace context, to inform robust trials assessing training efficacy and clinical behavior change.

Virtual reality (VR) as a training modality has the potential to address many of the current limitations in stroke workflow optimisation training. VR is immersive, engaging and increasingly used in professional training. Training content is applied and contextualized, without the risks or associated costs of on-the-job learning. Moreover, VR allows specialist knowledge and best-practice training to be deployed in a stand-alone VR headset, which can be standardized and available on-demand at hospital sites. VR delivery also allows for objective (and reportable) assessment of user performance and individualized user feedback. In a range of educational settings, VR-based training consistently increased student engagement and motivation (23, 24). Further, VR applications are effective at training practical skills (25, 26) and knowledge development (27–29). While VR has previously been applied in the context of both stroke management and medical education (30–32), applications for stroke have primarily targeted patients (e.g., for limb rehabilitation) rather than healthcare professionals [reviewed in (33, 34)]. To our knowledge, VR has not previously targeted healthcare professionals to support stroke management workflow training. While VR holds promise for addressing barriers to on-site healthcare workforce training, the acceptability and feasibility of this approach remain unclear. In particular, the healthcare workforce has limited experience/exposure to VR technology and deploying technology into complex healthcare settings can be difficult. A novel VR-based training module may not support clinical change, unless implementation and support strategies are clearly addressed. Given these concerns, research is required to determine the acceptability and feasibility of VR-based training in the context of stroke management in a real-world clinical setting.

We recently initiated the TACTICS—“Trial of Advanced CT Imaging and Combined Education Support for Drip and Ship” stepped-wedge cluster trial, which is assessing a “package intervention” to support advanced CT imaging and streamlined workflow training. As part of the educational component of the intervention we developed TACTICS VR, a novel VR-based training application to upskill healthcare professionals in optimal stroke workflow processes. The VR training application immerses users in a real-world stroke case to provide realistic on-site training in a hospital setting. In this case study, we outline the key features and elements of the TACTICS VR training platform and the critical considerations which informed its creation and design. We also report feedback from its pilot implementation within the broader TACTICS clinical trial framework into 7 Australian hospitals. The primary study aims were to (1) develop an evidenced-based VR training module for hyper-acute stroke workflow, (2) determine the feasibility of implementation in real-world hospital settings, and (3) capture trainee feedback on user experience, perceived usability, acceptability and utility.

## Materials and Methods

### Project Conception and Scoping

TACTICS VR was conceived through initial discussions between the Center for Advanced Training Systems (ATS; https://www.advancedtrainingsystems.org.au) and the TACTICS trial team in 2018. The TACTICS trial is a National Health and Medical Research Council (NHMRC)-funded, stepped-wedge clinical trial that aims to improve access to stroke reperfusion therapies, particularly at RRR hospital sites across Australia. The trial is evaluating the effectiveness of an education and training “package intervention” supporting implementation of advanced acute stroke imaging, streamlined workflow and protocols for reperfusion therapy delivery. The trial utilizes quality improvement methodologies and is based on the principles of the Theoretical Domains Framework (35). The trial will be implemented across 6 clusters in 3 Australian states in a stepwise design through 2022. The current manuscript reports data on TACTICS VR implementation into Cluster 1, which included 7 NSW hospitals.

### Content Scoping, Development, Review and Approvals

**Figure 1:**
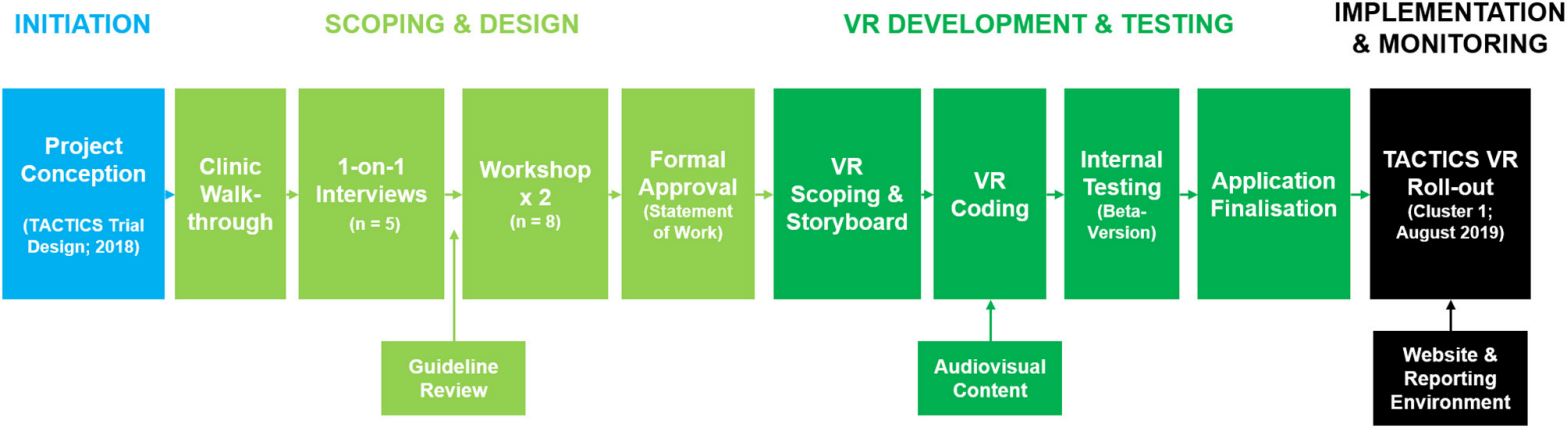
TACTICS VR development pathway. TACTICS VR application development occurred *via* a structured process from initiation, through scoping and design, VR content development, iterative rounds of internal testing and through to implementation and monitoring.

TACTICS VR was developed *via* a structured, stepwise approach (overview schematic; Figure 1). The process involved members from ATS, the TACTICS clinical trial team and subject matter experts (SMEs) drawn predominately from the Hunter New England Area Stroke Network with relevant stroke workflow expertise including specialists, nurses, and radiographers.

The overall concept and philosophy for TACTICS VR was provided by the TACTICS trial team and ATS managed scoping and specific content development. An initial “macro-level” overview of hyper-acute stroke management workflow was generated based on on-site clinic walkthroughs with an experienced stroke physician. This process identified key aspects of workflow procedures and contextual insights in a hospital setting. Reference photographs were taken to inform development of the virtual environments (e.g., ED, radiology suite and equipment). Information was then formulated into a brief outline, which was presented to SMEs in one-on-one meetings for feedback and approval (*n* = 3 stroke neurologists; 2 stroke nurses). Feedback highlighted common mistakes that can delay clinical workflow. Relevant stroke guidelines were also reviewed to ensure key recommendations were integrated [e.g., Australian Clinical Guidelines for Stroke Management (36)]. All feedback was integrated into a single statement of work, which was reviewed by the TACTICS clinical trial team at 2 workshops. The 1st workshop sought consensus on optimal workflow and a specific patient case to be presented. The 2nd workshop focused on identifying common sub-optimal workflow decisions, to inform user interactions/questions and assign time penalties for sub-optimal decisions. Supplemental resources were also sourced from SMEs, including short videos of workflow aspects and recordings of simulated hospital settings. The completed workflow was summarized into a single document and formally approved by members of the ATS, SMEs and the TACTICS trial team. All aspects of the approved statement of work were then mapped into a detailed decision-tree document, which was circulated to content developers for quotes.

### VR Application Development

Application coding proceeded *via* an iterative, step-wise approach including 4 phases: (1) VR scoping and concept creation; (2) content development and refinement; (3) internal beta-testing; and (4) application finalization. A software developer (Jumpgate VR, Adelaide, Australia) was contracted to code the TACTICS VR application for the Oculus Go headset using the Unity platform. Intellectual property relating to TACTICS VR is owned by The University of Newcastle. If interested in accessing the TACTICS VR application for research purposes, please contact the corresponding author.

### Features of TACTICS VR—A Hyper-Acute Stroke Management Workflow Training Application

The TACTICS VR application consists of a fully computer-generated imagery (CGI) environment, providing a first-person virtual walkthrough of a single stroke patient case from the doctor/stroke team leader's perspective. It was developed for use by a variety of medical staff including junior medical officers (JMOs), nurses, radiographers and Emergency Department staff. The primary target audiences were identified as JMOs and physicians in RRR sites with limited experience treating stroke patients. Recognizing that healthcare professionals are time-poor, the total training delivery time was restricted to 20–30 min. Consideration was made to streamline content delivery within this timeframe, emphasizing critical workflow aspects rather than basic knowledge development.

**Figure 2:**
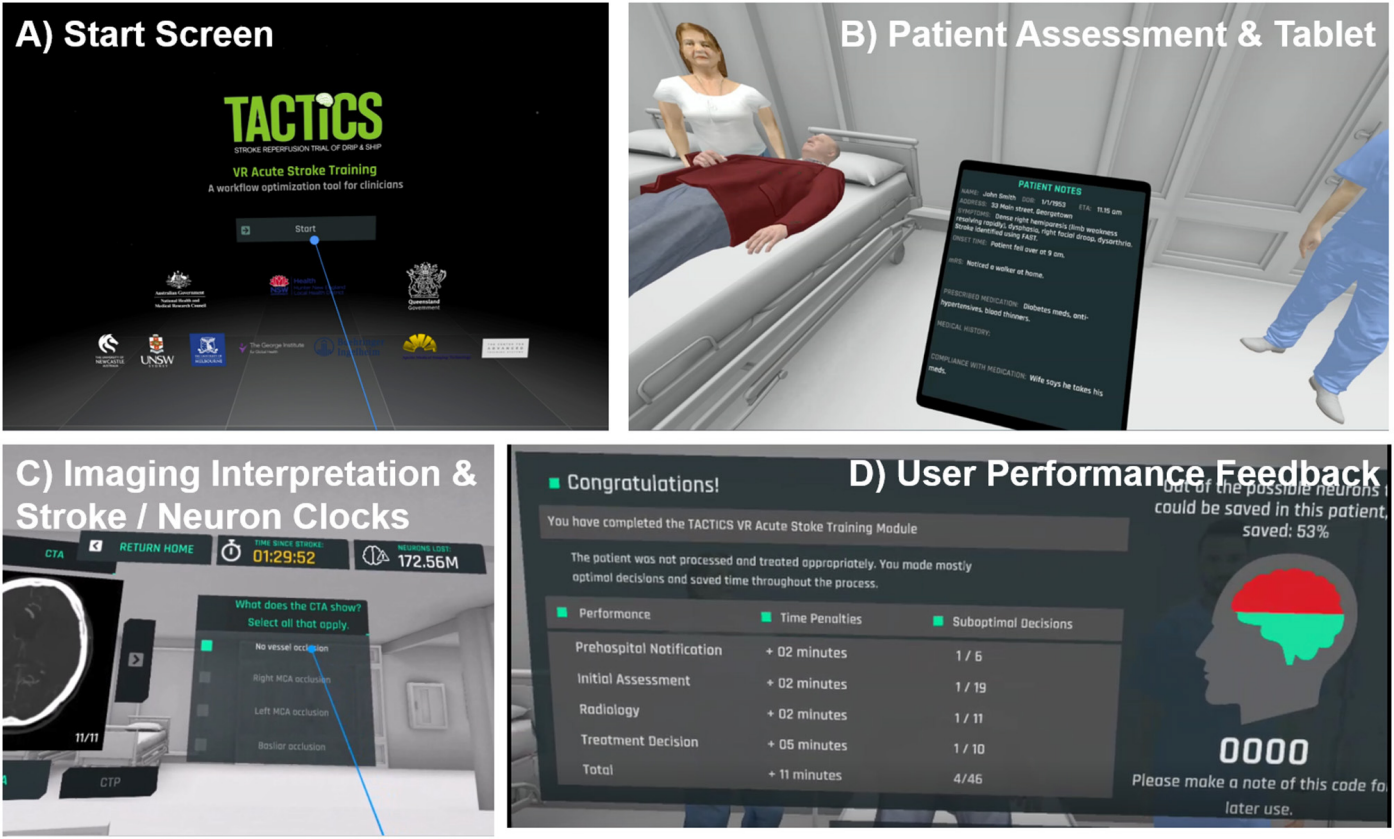
Screenshots and key features of TACTICS VR. **(A)** starting scene, **(B,C)** representative scenes, user interactions and **(D)** performance feedback.

The application places users into a generic, intentionally plain, virtual hospital which presents a non-site-specific environment with limited visual distractions (Figure 2). The use of a minimalistic design and CGI allows delivery across a range of clinical settings, and has the greatest potential for user interaction and compatibility with future content modifications. As hospitals are typically noisy, low-level background audio was included to provide realism. Educational content is presented in pop-up text, audio and video formats (Figures 2B,C). Content is delivered *via* an interactive interface, to promote active learning and trainee engagement. Users query semi-realistic virtual avatars, interact with the virtual environment (e.g., phone, computer) and respond to knowledge-testing elements (e.g., multiple-choice questions). To highlight the need for accurate but efficient decision-making, users are provided with immediate feedback on their decisions. To specifically emphasis the time-critical nature of the workflow a “stroke clock” and “neuron clock” were integrated, which are visible throughout the module. These features were suggested during initial internal testing by stroke clinicians, to add a gamification element to the training and to emphasize the real-world consequences of sub-optimal stroke management decisions. Suboptimal user decisions result in time penalties adding time to the stroke clock whilst the neuron clock provides an estimate of neuronal loss, increasing with both time in training and penalties for sub-optimal decisions. Penalties approximate the real-world delays that each sub-optimal decision would have on stroke management workflow and treatment.

Upon starting TACTICS VR, users are initially oriented to the environment, introduced to the stroke- and neuron clock as well as the virtual notepad/tablet, which displays collected patient information. Users proceed through a linear narrative, including: (1) opening title screen/introduction, (2) pre-hospital notification, (3) initial assessment, (4) radiology/imaging, (5) treatment and consent, and (6) user feedback (Table 1). Each of the 4 clinical scenes (items 2–5 above) address specific learning objectives and critical areas for skill development. All clinical scenes feature an introduction (e.g., talking-head video), relevant clinical information, user interactions (e.g., multiple choice questions, avatar interaction) and tailored guidance/feedback triggered by user responses. A summary of overall training performance and outcomes is provided to users in the headset upon completion (and transmitted automatically *via* WiFi, as detailed below; Figure 2D).

**Table 1:** TACTICS VR scenes, key features and user interactions.

**Scene**	**Features**	**User interactions**
Start scene/introduction	• VR infinity space • Contributors/funding bodies • Overview of TACTICS VR • Demographics capture (hospital) • Orientation to TACTICS VR (tablet, stroke/neuron clock) • Initiation of training	• Navigation buttons • Q&A = Demographics
Pre-hospital notification	• ED desk + phone • Initial patient data capture • Pre-arrival workflow priorities	• Phone interaction • Q&A = Patient details, workflow
Initial assessment	• ED bed • Detailed patient data capture • Team-based workflow and parallel processing • Assessment workflow priorities	• Avatar interactions = nurse, ambulance attendant, next-of-kin • Q&A = Patient details, workflow
Radiology/imaging	• Radiology department + ED • Advanced imaging capture • Preliminary imaging interpretation • Imaging/treatment workflow priorities	• Avatar interactions = CT radiographer • Computer interaction = imaging interpretation • Q&A = Imaging selection/interpretation, treatment eligibility, workflow
Treatment and consent	• Resuscitation/treatment suite • Treatment consent & delivery	• Avatar interactions = nurse, next-of-kin • Q&A = Consenting, treatment eligibility and delivery
Feedback	• User performance = time spent, penalties/estimated neuron loss, optimal response % (total/per scene)	

*CT, computed tomography; ED, emergency department; Q&A, question and answer*.

### Development of the Broader TACTICS VR Training Platform

Beyond the stand-alone VR-based application, we also developed additional tools as part of a broader TACTICS VR training environment. This included an end user-facing website (www.tacticsvr.com) and automated reporting environment, which were developed by external web developers (HyperWeb, Newcastle, Australia; Jumpgate Virtual Reality, Adelaide, Australia).

**Figure 3:**
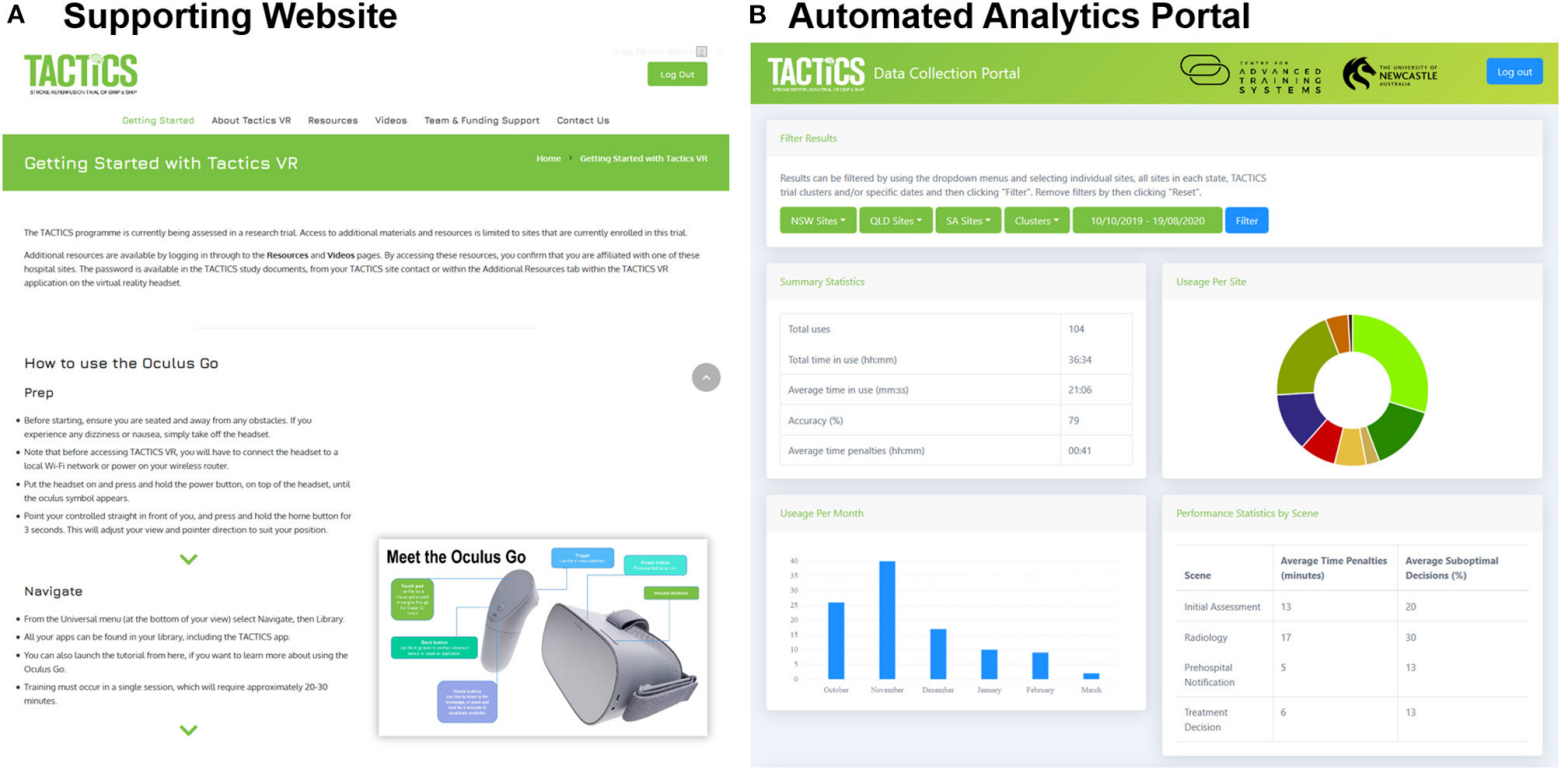
TACTICS VR supporting training environment. The TACTICS VR integrates. **(A)** A supporting website providing user support and educational resources and **(B)** automated back-end data analytics portal for capture of training uptake and user performance.

The user-facing website provides access to additional resources and supporting information including an access guide, information about the TACTICS VR application, the overall TACTICS clinical trial, study team, funding organization details and contact information (screenshot Figure 3A). The website also hosts password-protected resources to support the TACTICS clinical trial team, including clinical trial documents, detailed user guides, workshop presentations and educational videos.

The automated analytics portal was designed as a back-end reporting database for data capture, collation and presentation (screenshot Figure 3B). The portal is password-protected and access restricted to members of the study team. Data (including sessions, locations, and performance) is automatically transmitted from the VR headset *via* WiFi to a study database. Performance data captures each user interaction throughout the training module (e.g., question and answer responses) and the accuracy of the interaction (i.e., correct vs. suboptimal decisions). Data can be filtered by date, hospital sites and/or clinical trial cluster. Individual user reports are accessible, providing information on the time spent in training, responses to individual questions and compiled user performance data for each scene and overall. Data can be exported in both pdf and csv format to support analysis, reporting of research findings and real-time monitoring of usage to inform ongoing implementation.

### Implementation of the TACTICS VR Application

The current manuscript reports data from Cluster 1 implementation of the TACTICS clinical trial, consisting of 7 hospitals. The study protocol was approved by the Hunter New England Health Human Research Ethics Committee (REGIS Ref 2019/ETH01238; HNEHREC Ref 18/09/19/4.13) and lodged with the University of Newcastle Human Research Ethics Committee (H-2019-0343). Prior to deployment of TACTICS VR training, all study sites provided consent to participate. Individual user participation was voluntary and users were able to use TACTICS VR without participating in the study. Per protocol, participants were advised that consent was implied by completion of the surveys.

#### Study Participants and Recruitment

TACTICS trial site coordinators were initially briefed and trained with the VR headset at the beginning of cluster deployment at a face-to-face workshop. Site coordinators were then encouraged to recruit relevant staff at their hospital to use TACTICS VR throughout the 3-month active implementation phase of the trial. Coordinators were encouraged to make training available to any interested healthcare professionals at their site, in particular members of the stroke team, emergency department and radiology/imaging. Regular monthly teleconferences with site coordinators occurred over this phase, as an opportunity for updates and feedback on implementation of the overall TACTICS trial intervention. At meetings, attendees were provided with updates on TACTICS VR usage and asked to provide feedback on any issues/barrier identified. Recruitment for the current study was not specifically raised at sessions.

#### Study Protocol

Participants were invited to complete paper surveys before (pre-survey) and after (post-survey) training with the TACTICS VR application. Surveys were drafted with input from research team members as relevant for subsequent adoption of the training approach within the local health district informed by clinical experience. Surveys were streamlined in recognition of the severe time constraints of the targeted healthcare professional audience. Pre-survey questions included relevant demographics (e.g., user specialty area, employment category, previous stroke management experience and VR experience), general expectations of VR-based training, anticipated learnings and confidence in stroke management (full question list and participant responses in Supplementary Table I). Post-survey questions sought feedback on TACTICS VR (e.g., hardware comfort, design, user interactions, feedback and educational content), areas for improvement and follow-up questions on confidence in stroke management (Supplementary Table II). As noted above, user training performance data was automatically transmitted when users completed VR training *via* WiFi from the VR headset to a dedicated database.

### Statistical Analysis

Statistical analysis was performed using Prism v.8 (GraphPad, USA). All data is presented as mean ± SD. Comparisons between responses to the two repeated pre- and post-survey questions were analyzed using t-tests, adjusted for multiple comparisons. *P* < 0.05 were considered statistically significant for all analyses.

### Development and Implementation Costs

Direct costs associated with development of the TACTICS VR training platform included: VR application coding (~$35,000 AUD); design, development and integration of the automated analytics portal (~$16,000 AUD); user-facing website design and development (~$4,000 AUD) and hardware (VR headset, WiFi router; ~$500 AUD/site). Additional costs associated with project management, application testing, research assessment, ongoing support and maintenance and implementation within the broader TACTICS clinical trial were not specifically captured.

## Results

### TACTICS VR Usage

TACTICS VR was rolled-out to Cluster 1 of the TACTICS clinical trial, which included a comprehensive stroke hospital hub site and 6 spoke sites in rural/regional settings. A total of 104 TACTICS VR sessions were logged between October 2019 and March 2020. Monthly session numbers increased from October (26 sessions) to November (37), then gradually decreased through early 2020 (December = 17; January = 10; February = 9, March = 2).

Usage sessions were logged at all 7 hospital sites, including 15 sessions at the hub hospital and 21, 15, 13, 8, 5 and 3 sessions at each of spoke sites. Location demographics were not initially integrated at trial initiation, so the first 31 sessions were captured without this information. The mean VR session time was 21 min 06 s ± 8 min 7 s (mean ± SD), equating to a total training time of 36 h 34 min across all 104 user sessions. User response accuracy was 79 ± 12% overall, resulting in an average of 41 ± 33 min per session in time penalties. User accuracy was highest for the pre-hospital notification (87%; 5 total scene decisions) and treatment scenes (87%; 3 decisions) and lower in the initial assessment (80%; 8 decisions) and radiology scenes (70%; 8 decisions).

### TACTICS VR User Survey Feedback

All TACTICS VR users were invited to complete paper-based surveys before and after training. A total of 61 pre-training and 58 post-training survey responses were received. Respondent demographics are presented in Table 2 and a complete list of survey questions and responses are provided in Supplementary Tables I, II. Survey responses were received from the hub hospital and 5 of 6 spoke hospital sites (Table 2). Respondents worked in a range of specialty areas, including Emergency Care (*n* = 27), Acute Stroke/Neurology (11), Radiology (11) and Intensive Care (2). The majority of respondents identified as nurses (24) or doctors (21), with 12 as “other,” which included 2 in allied health, 2 radiographers and 1 student registered nurse. Respondents had a range of previous experience in stroke management, with 15 indicating experience caring for/treating ≤10 patients (designated “low experience” by the research team, including expert neurologists) and 21 >40 patients (“high experience”; Table 2). Seventeen respondents indicated they were prone to motion sickness, when asked a binary yes/no question (no = 43; Table 2).

**Table 2:** Survey respondent demographics.

**Query**	**Response (*n*)**
Hospital site	Hub hospital site (pre = 11/post = 11) Spoke site 1 (13/13) Spoke site 2 (12/12) Spoke site 3 (9/9) Spoke site 4 (10/9) Spoke site 5 (6/4) Spoke site 6 (0/0)
Specialty area	Emergency care (27) Radiology (11) Acute stroke/neurology (11) Intensive care (2) Other (8)
Employment category	Doctor (21) Nurse (24) Other (12): • Allied health (2) • Radiographer (2) • Student RN (1)
Experience with stroke treatment	≤10 patients (15) 11–20 (7) 21–30 (8) 31–40 (7) >40 (21) Not applicable (3)
Experience with VR technology	None (36) ≤10 total h (21) 10–49 h (2) 50–99 h (1) ≥100 h (0)
Prone to motion sickness	Yes (17) No (43)

*RN, registered nurse; VR, virtual reality; n = 61 total pre-training survey respondents (n = 58 post-training)*.

When asked about their previous experiences with stroke management workflow, respondents reported relatively positive overall experiences assessing or treating acute stroke patients (mean ± SD = 4.0 ± 0.8; strongly agree = 5, strongly disagree = 1). Further, before completing VR training they reported high levels of confidence in a number of areas including: ability to effectively assess and treat stroke patients (3.9 ± 1.0), optimally communicate with colleagues to enable effective treatment (4.3 ± 0.7), ability to determine appropriate treatment (3.8 ± 0.7), knowledge around accessing telehealth or senior colleague support (4.0 ± 0.8), understanding acute stroke workflow practices (4.0 ± 0.8) and ability to make improvements to acute stroke care provision (3.6 ± 0.9).

Respondents had relatively little lifetime experience with VR technology; 36 with no previous experience, 21 ≤10 total hours, 2 10–49 h, and 1 50–99 h (Table 2). Despite limited VR exposure, respondents predominantly agreed with the statement that “VR can be an effective method to teach or transfer knowledge about acute stroke workflow practices” (mean ± SD = 3.69 ± 0.78; strongly agree = 5, strongly disagree = 1) and were moderately confident in their ability to manage technical aspects of VR training (3.47 ± 1.07). Respondents believed *a priori* that VR technology would be most useful for development of procedural knowledge (*n* = 46), communicating and transferring knowledge about workflow (28), intra-professional communication (20) and “other” areas (6). Only 1 respondent indicated they did not believe VR would be useful in any of these areas.

**Figure 4:**
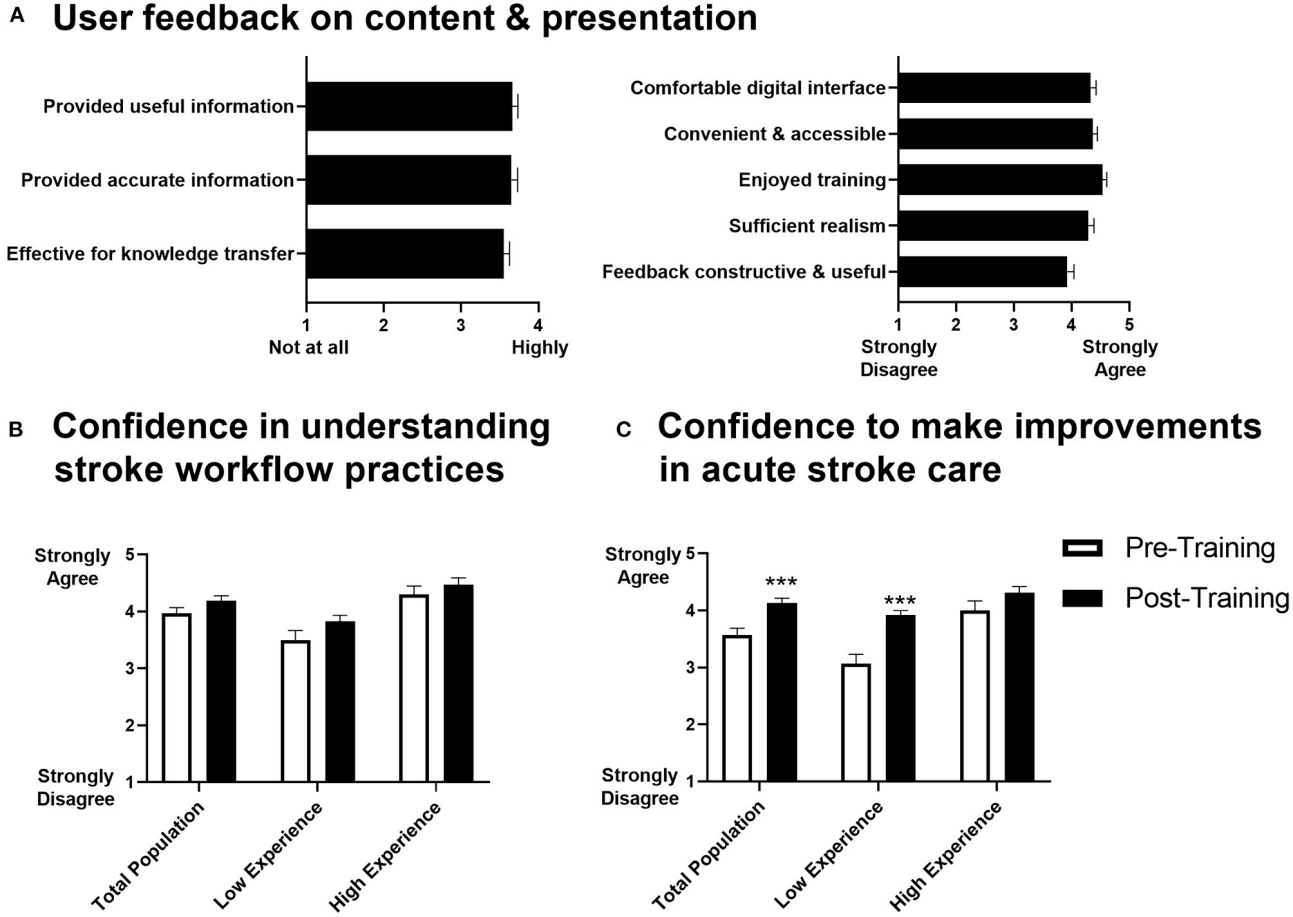
User survey feedback on TACTICS VR. **(A)** User feedback on content and presented information, **(B)** user confidence in understanding of stroke workflow practices and **(C)** user confidence to make improvements in acute stroke care (*n* = 53-58; *n* = 14 “low experience” = ≤10 patients cared for/treated; *n* = 18 “high experience” = >40 patients cared for/treated; data presented as mean +/– SEM; ***p < 0.001).

After completing TACTICS VR training, respondents provided feedback on aspects of hardware and software design, content, educational value and implementation approach. Users indicated that TACTICS VR provided both useful and accurate information, effective knowledge transfer, was comfortable, convenient and accessible, they enjoyed training, it was sufficiently realistic and that feedback was constructive and useful (mean scores ranged from 3.9 to 4.7; 1 = strongly disagree, 5 = strongly agree; Figure 4A). Further, respondents agreed that training provided an awareness of certain elements of intrapersonal communication that are likely to be useful (4.4 ± 0.6) and were confident in their ability to transfer knowledge gained during the VR training into practice (4.3 ± 0.6). They agreed with the statements that VR is an effective method to teach or transfer knowledge about acute stroke workflow (4.4 ± 0.6) and would be effective for other clinical and medical professionals in other areas of practice (4.5 ± 0.5). The simulation was deemed appropriate to their level of knowledge and skills (3.9 ± 1.0), allowed the opportunity to understand prioritization around stroke assessment and acute care practices (4.4 ± 0.5) and allowed users to analyse their own behavior and actions to reflect on how they can improve performance in the future (4.2 ± 0.6). We note that due to study design, user feedback on VR training could not be compared to alternative training modalities.

In comparison to pre-training responses, we noted a trend toward increased confidence in stroke workflow practices (Figure 4B) and respondents were more confident in their ability to make improvements in stroke management (post-training mean ± SD = 4.1 ± 0.6; pre-training = 3.6 ± 0.9; Figure 4C). This improvement was most prominent in the respondents with less experience managing stroke (i.e., those that indicated the lowest level of experience in caring for/treating stroke patients in the pre-training survey [≤10 patients]; post = 3.9 ± 0.3; pre = 3.1 ± 0.6; Figure 4C). Thirty-nine of 52 respondents reported that TACTICS VR training contributed toward plans to improve acute stroke care, with 31 planning to make changes and 8 already making changes. Ten of 54 respondents reported that they experienced motion sickness and/or nausea during training in the post-training survey; 4 discontinued training as a result, while 6 were able to complete training. Only 3 of the affected respondents had indicated they were prone to motion sickness prior to training.

When asked in open-ended questions what elements of the TACTICS VR training module were most beneficial, user responses primarily related to TACTICS VR overall, design aspects, the VR format and stroke-specific education outcomes. Selected comments are provided below, which have been selected to be representative and highlight the main themes raised. All responses are provided in Supplementary Table II. Respondents indicated the most beneficial elements as:

- “*Concise, direct and relevant”*
- “*Engaging and realistic”*
- “*Multidisciplinary interaction”*
- “*Real case simulation. Excellent interface”*
- “*Explanations of rationale for certain aspects of care”*
- “*Feedback on timing/decision making”*
- “*Good summary of acute stroke processes”*
- “*Take home point for me* = *don't waste time, keep moving and intervening”*
- “*Emphasis on workflow/avoiding preventable delays.”*

Respondents were also asked what elements of TACTICS VR could be improved upon. Several specifically responded that there were no areas for improvement. Other respondents identified aspects relating to VR technology familiarity, site-specific content design and areas for future development/expansion. Comments included:

- “*Further detail added to virtual environment to mirror real world”*
- “*Prompting of what to do next to make it more time effective”*
- “*Once familiar with VR training—repeated practice would be beneficial”*
- “*Difficult to attain best results when unfamiliar with the technology”*
- “*Would like more information about the errors that were made”*
- “*Variety of cases to emphasize different problems (e.g., contraindications to thrombolysis, BP control)”*
- “*Different paths for different roles (e.g., when the CT interpretation is happening, maybe focus on nursing assessment and monitoring during the scan rather than interpreting the CT)”*
- “*Incorporate telestroke call as big part of process for us, more detail re obtaining consent, findings on CT a bit unclear—I made a guess based on clinical presentation.”*

## Discussion

This report describes the initiation, concept design, pilot implementation and feasibility assessment of TACTICS VR, the first VR-based application specifically developed to train healthcare professionals in hyper-acute stroke management workflow. The VR headset-based training module is supported by a user-facing supporting website and back-end reporting environment to facilitate automated usage data capture. The TACTICS VR training platform was developed to address an identified training gap and deployed as part of the broader TACTICS clinical trial and is a key intervention element in the ongoing trial which will be completed in late 2022. In this study, we evaluated the usability, acceptability and utility of the VR-delivered stroke workflow optimisation training across 7 NSW hospitals. Feedback indicated a high level of usability and acceptability amongst the target population and the feasibility of deploying VR training into a real-world clinical workplace setting. Users provided positive feedback on delivery, design and knowledge development including increased self-reported confidence to make improvements in acute stroke care.

Current healthcare professional training is typically delivered face-to-face by a trainer with content-specific expertise, *via* traditional teaching modalities (e.g., lectures, printed materials, PowerPoint presentations). This approach supports the delivery of evidence-based, clinically-relevant educational content, but is expensive to deliver and difficult to scale particularly in RRR settings. Group education approaches can contribute to relatively small improvements in professional practice (e.g., <10%) (38) and are better than passive delivery of clinical guidelines and recommendation. However, less than two-thirds of Australian physicians and nurses involved in stroke management report having received any interactive or competency-based training (15). Limited studies have assessed simulation-based approaches to support stroke workflow training (39). We included an extensive scoping phase in the development of TACTICS VR with input from expert stroke clinicians to ensure content was evidence-based, accurate and relevant and tailor delivery to specific user needs. TACTICS VR users confirmed that the resulting content was useful and accurate, increased their confidence and provided constructive feedback. We acknowledge that TACTICS VR was delivered in the context of a broader education implementation trial, and consideration of the local hospital environment, staffing and resources will be critical to enact major change in patient outcomes. Our data demonstrate that TACTICS VR training is feasible and acceptable within this context. Additional research is now required, which applies robust study design to assess training efficacy, effects on clinical behavior and compares this approach to alternative training modalities.

VR-based training suits scalable, standardized and on-site training. This flexibility is particularly relevant for RRR settings. The efficacy of individual VR training applications varies depending on the specific training context and learning outcomes assessed. Assessments of VR training tools most consistently identify improvements in trainee engagement (23, 24) and procedural training (25, 26), two key unmet needs in stroke workflow training. To promote user engagement and maximize the benefits of using VR technology, TACTICS VR was specifically designed to promote user interactions and active learning (e.g., interactive elements, gamification). This was balanced by the recognition that healthcare professionals are time-poor, and content inclusion emphasized critical workflow information delivered in a total training time of 20–30 min. Survey responses indicate that the VR approach was well-received, despite limited knowledge and experience with the technology. Users indicated that hardware was comfortable, convenient, accessible, enjoyable and sufficiently real for training.

A range of factors are recognized to improve stroke reperfusion treatment outcomes including expert multidisciplinary care, individual and team-based training, streamlined systems of care and clinician experience/confidence (40). Implementation trials to improve stroke management workflow are often complex and resource-intensive. For example, in our TIPS trial testing a complex systems intervention to support increased thrombolysis implementation we included integration of “change champions,” situational analysis, goal-setting, comparative feedback, active education and collaboration (19, 20). Despite the resource-intensity of this complex multi-faceted intervention, the trial failed to sustain enduring change in IVT treatment rates (despite a small temporary improvement), suggesting that additional and ongoing support is likely to be required for sustained change (19). Additional trials implementing similarly complex interventions have reported modest increases in IVT treatment uptake (37, 41, 42). These trials also highlight the complexity and resource-intensiveness of strategies to alter clinical behavior and improve delivery of IVT treatment alone, without even addressing other aspects of stroke workflow (e.g., advanced imaging, endovascular thrombectomy, and stroke rehabilitation). These approaches are particularly difficult to implement in RRR settings. We acknowledge this complexity and note that in the current research setting TACTICS VR training implementation was but one element of a more complex research trial. Pilot implementation of TACTICS VR training at 7 NSW hospitals did identify several aspects that could be improved. Early in implementation, we identified issues with WiFi connectivity across multiple hospital IT systems. This issue was overcome by supplying mobile WiFi routers to maintain connectivity. Further, TACTICS VR deployment was supported by on-site trial coordinators at each hospital site. In informal interviews, site coordinators suggested that additional implementation/deployment approaches could increase training reach (e.g., deployment in local EDs and integration into existing clinical training programs). These factors will be further explored in future studies, to assess mechanisms that support long-term integration and sustainment of VR training.

We note several limitations which should be considered when interpreting data from this study. As noted, user recruitment was led by on-site trial coordinators to target relevant trainees involved in stroke management. As such, participant selection was non-random. An advantage of this approach was the recruitment of a broad range of multi-disciplinary healthcare professionals who provided diverse feedback on TACTICS VR training. Implementation was limited to 7 NSW hospitals, which may not be representative of stroke training and management in all settings. The sites did include rural and regional hospitals, which were the primary intended audience for TACTICS VR. The ongoing TACTICS trial will roll-out TACTICS VR training across a further 5 study clusters in 3 Australian states. We note that TACTICS VR training was deployed as part of a broader educational package in the TACTICS trial, which has a stepped-wedge study design. TACTICS VR training is intended to complement existing training approaches and resources, as a valuable add-on approach that can be deployed on-site, including in RRR settings. Due to constraints in study design, no control group could be included in the current study and we are unable to compare the effects of TACTICS VR training to alternative training approaches. Additional research comparing TACTICS VR to alternative training modalities would be useful to inform optimal approaches. To this end, we are currently undertaking investigations directly comparing different delivery formats for training (e.g., 2D vs. VR-based training). Additional assessment using validated tools would also be of value [e.g., usability *via* System Usability Scale (43), Post-Study Usability Questionnaire (44); immersion/presence *via* Presence Questionnaire (45)]. Metrics on clinical behavior and patient outcomes are being captured in the broader TACTICS clinical trial, which will provide insights into the effects of the overall intervention but will not allow assessment of the direct effect of TACTICS VR specifically. Additional assessment is required that provides precise data on training uptake and the cost vs. benefit of the VR training approach compared to other training modalities.

User feedback on TACTICS VR training was overall very positive and included suggestions for development of additional content. Specifically, a VR user tutorial, multiple/additional healthcare perspectives, detailed stroke assessment tasks and expanded workflow areas were suggested. This feedback has directly led to subsequent scoping and development of a dedicated stroke telehealth expansion module and additional planned modules from different healthcare professional perspectives (e.g., nursing, pre-hospital/ambulance). Further research is now required to identify effects of TACTICS VR training on clinical practice and ultimately patient outcomes. This data will be forthcoming from the TACTICS trial. Economic analysis would be useful to compare existing training approaches to VR-based deployment. Further research is also required to identify optimal implementation strategies for TACTICS VR training (e.g., hospital department, location, staffing, integration into existing IT infrastructure and training programs).

## Data Availability Statement

The original contributions presented in the study are included in the article/Supplementary Material, further inquiries can be directed to the corresponding author/s.

## Ethics Statement

The studies involving human participants were reviewed and approved by Hunter New England Health Human Research Ethics Committee (REGIS Ref 2019/ETH01238; HNEHREC Ref 18/09/19/4.13) University of Newcastle Human Research Ethics Committee (H-2019-0343). Written informed consent for participation was not required for this study in accordance with the national legislation and the institutional requirements.

## Author Contributions

RH, EN, CP, NS, CL, and FW contributed to conception and design of the study. RH, AK, MK, EN, AR, MP, CP, CG-E, NS, CL, and FW contributed to scoping and content development for the virtual reality application. RH, SM, AK, AR, and MC contributed to data collection, analysis, and interpretation. RH, SM, and FW wrote the first draft of the manuscript. All authors contributed to manuscript revision, read, and approved the submitted version.

## Funding

Funding support was provided for the TACTICS—Trial of Advanced CT Imaging and Combined Education Support for Drip and Ship from the Australian National Health and Medical Research Council (NHMRC). The TACTICS trial is also funded *via* the University of Newcastle, Queensland Department of Health, Apollo Medical Imaging Technology Pty Ltd, Boehringer Ingelheim, and the Hunter New England Local Health District (HNELHD). Funding for the supporting website and reporting environment was provided by the Priority Research Center for Stroke and Brain Injury (the University of Newcastle) *via* Medical Research Support Program (MRSP) infrastructure and Joint Research Engagement (JRE) funding allocations and new protocol development *via* a NSW Regional Health Partners Rapid Applied Research Translation grant. The funders were not involved in the study design, collection, analysis, interpretation of data, the writing of this article or the decision to submit it for publication.

## Conflict of Interest

All authors were involved in the development of the TACTICS VR application. Intellectual property for the TACTICS VR training platform is owned by The University of Newcastle (Australia).

## Publisher's Note

All claims expressed in this article are solely those of the authors and do not necessarily represent those of their affiliated organizations, or those of the publisher, the editors and the reviewers. Any product that may be evaluated in this article, or claim that may be made by its manufacturer, is not guaranteed or endorsed by the publisher.
