# Supplementary material for: Development and Pilot Implementation of TACTICS VR: A Virtual Reality-Based Stroke Management Workflow Training Application and Training Framework
Source: Front Neurol. 2021 Nov 11;12:665808. doi: 10.3389/fneur.2021.665808 (PMC8631764; doi:10.3389/fneur.2021.665808)
Supplement: Supplementary file 1 [file Data_Sheet_1.DOCX]

# Supplementary Materials Contents

**Supplementary Table I: Pre-training user survey questions and participant responses**

**Supplementary Table II: Post-training user survey questions and participant responses**

# Supplementary Tables

**Supplementary Table I: Pre-training user survey questions and responses**

| **Question** | **Response options (n)** |
| --- | --- |
| Hospital Site | Hub hospital site (11)  Spoke site 1 (13)  Spoke site 2 (12)  Spoke site 3 (9)  Spoke site 4 (10)  Spoke site 5 (6)  Spoke site 6 (0)  **Note – site names have been de-identified for public reporting due to ethics considerations* |
| What is your specialty area of work at this hospital? | Interventionist Care  Emergency Care (27)  Radiology (11)  Acute Stroke / Neurology (11)  Intensive Care (2)  Stroke Rehabilitation  Other (8) |
| What category best describes your employment at this hospital? | Doctor (21)  Nurse (24)  Nurse Practitioner  Other (12):   - Medical Student - Allied Health (2) - Radiographer (2) - Student RN (1) - Stroke Coordinator |
| How many acute stroke patients have you cared for or treated over the course of your career (estimated) | ≤10 patients (15)  11-20 (7)  21-30 (8)  31-40 (7)  >40 (21)  Not applicable (3) |
| My overall experience with assessing or treating acute stroke patients over the course of my career has been positive | Strongly agree  Agree  Undecided  Disagree  Strongly disagree  Not applicable  (Mean = 3.97 +/- 0.75; Strongly agree = 5) |
| I am confident in my ability to effectively assess and treat stroke patients | All of the time  Some of the time  Undecided  Rarely  Never  Not applicable  (Mean = 3.92 +/- 0.95; All of the time = 5) |
| I am confident in my ability to optimally communicate with my colleagues to enable effective treatment of acute stroke patients | All of the time  Some of the time  Undecided  Rarely  Never  Not applicable  (Mean = 4.30 +/- 0.71; All of the time = 5) |
| I am confident in my ability to determine appropriate treatment(s) for acute stroke patients | All of the time  Some of the time  Undecided  Rarely  Never  Not applicable  (Mean = 3.84 +/- 0.70; All of the time = 5) |
| I am confident in my knowledge around accessing telehealth or senior colleague support for acute stroke assessment or treatment | Strongly agree  Agree  Undecided  Disagree  Strongly disagree  Not applicable  (Mean = 4.00 +/- 0.78; Strongly agree = 5) |
| I am confident in understanding of workflow practices for acute stroke assessment and treatment | Strongly agree  Agree  Undecided  Disagree  Strongly disagree  Not applicable  (Mean = 3.97 +/- 0.75; Strongly agree = 5) |
| I am confident in my ability to make improvements to how acute stroke care is provided to patients presenting to this hospital | Strongly agree  Agree  Undecided  Disagree  Strongly disagree  Not applicable  (Mean = 3.57 +/- 0.90; Strongly agree = 5) |
| What is your experience with virtual reality technology? | None (36)  ≤10 total hours (21)  10-49 hours (2)  50-99 hours (1)  ≥100 hours (0) |
| I believe that VR can be an effective method to teach or transfer knowledge about acute stroke workflow practices | Strongly agree  Agree  Undecided  Disagree  Strongly disagree  Not applicable  (Mean = 3.69 +/- 0.78; Strongly agree = 5) |
| I believe that VR would be most useful for (select all that apply) | Procedural knowledge (46)  Intra-professional communication (20)  Communicating and transferring knowledge about workflow (28)  None of the above (1)  Other (free-text; 6) |
| I feel confident in regards to managing the technical aspect of the VR training tool | Strongly agree  Agree  Undecided  Disagree  Strongly disagree  Not applicable  (Mean = 3.47 +/- 1.07; Strongly agree = 5) |
| Are you prone to motion sickness? | Yes (17)  No (43) |
| What do you hope to learn from the TACTICS Virtual Reality Acute Stroke training module? | Free-text |

VR = virtual reality; Data presented as mean +/- SD.

**Supplementary Table II: Post-training user survey questions and responses**

| **Question** | **Response options (n)** |
| --- | --- |
| To what extent has the VR training module provided useful information on optimal and suboptimal stroke workflow practices? | Highly useful  Mostly useful  Somewhat useful  Not useful  (Mean = 3.66 +/- 0.55; Highly useful = 4) |
| To what extent has the VR training module provided accurate information around optimal and suboptimal stroke workflow practices? | Highly accurate  Mostly accurate  Somewhat accurate  Not accurate  (Mean = 3.65 +/- 0.58; Highly accurate = 4) |
| To what extent did you find the VR training module an effective tool for transferring knowledge around acute stroke workflow practices? | Highly effective  Mostly effective  Somewhat effective  Not effective  (Mean = 3.55 +/- .57; Highly effective = 4) |
| The VR module has provided an awareness of certain elements of intrapersonal communication that are likely to be useful for trainees and staff in the management of acute stroke | Strongly agree  Agree  Undecided  Disagree  Strongly disagree  (Mean = 4.41 +/- 0.57; Strongly agree = 5) |
| I am confident that I can transfer knowledge obtained during the VR module into actual practice | Strongly agree  Agree  Undecided  Disagree  Strongly disagree  (Mean = 4.33 +/- 0.55; Strongly agree = 5) |
| I felt comfortable using the digital interface that guided me through the VR training module | Strongly agree  Agree  Undecided  Disagree  Strongly disagree  (Mean = 4.33 +/- 0.67; Strongly agree = 5) |
| I believe that VR is an effective method to teach or transfer knowledge about acute stroke workflow practices | Strongly agree  Agree  Undecided  Disagree  Strongly disagree  (Mean = 4.35 +/- 0.55; Strongly agree = 5) |
| I have found using the TACTICS VR equipment available to me convenient and accessible | Strongly agree  Agree  Undecided  Disagree  Strongly disagree  (Mean = 4.36 +/- 0.62; Strongly agree = 5) |
| I believe VR could be an effective method of teaching / knowledge transfer for clinical and medical professionals in other areas of practice | Strongly agree  Agree  Undecided  Disagree  Strongly disagree  (Mean = 4.47 +/- 0.54; Strongly agree = 5) |
| I have enjoyed the TACTICS VR training program | Strongly agree  Agree  Undecided  Disagree  Strongly disagree  (Mean = 4.53 +/- 0.57; Strongly agree = 5) |
| The simulation was appropriate for my specific level of knowledge and skills | Strongly agree  Agree  Undecided  Disagree  Strongly disagree  (Mean = 3.91 +/- 1.02; Strongly agree = 5) |
| The simulation allowed me the opportunity to understand prioritisation around stroke assessment and acute care practices | Strongly agree  Agree  Undecided  Disagree  Strongly disagree  (Mean = 4.36 +/- 0.52; Strongly agree = 5) |
| The simulation was of sufficient realism to communicate the critical aspects of workflow | Strongly agree  Agree  Undecided  Disagree  Strongly disagree  (Mean = 4.29 +/- 0.69; Strongly agree = 5) |
| The feedback provided at the end of the VR training module was constructive and useful | Strongly agree  Agree  Undecided  Disagree  Strongly disagree  (Mean = 3.92 +/- 0.85; Strongly agree = 5) |
| The simulation allowed me to analyse my own behaviour and actions, and to reflect on how I can improve my performance in the future | Strongly agree  Agree  Undecided  Disagree  Strongly disagree  (Mean = 4.21 +/- 0.60; Strongly agree = 5) |
| What elements of the TACTICS VR training module have you found most beneficial to your learning? | Free-text responses:   - All of it - Concise, direct and relevant - Engaging and realistic - The entire VR module. Realistic situations - The ability to cover large areas of information without being caught up in detail - Realistic time management - Multidisciplinary interaction - Visualisation and prioritisation of workflow - The immersion in the scenario and 3D aspects - The simulation is not awkward. It is like you are in the situation having to deal with what is presented calmly - The immersion in the environment / background noise / realism make this a better education format. “Push it yourself” – Love it! - Simulation of real life case - Real case simulation. Excellent interface - Explanations of rationale for certain aspects of care - Explanations of my errors - Feedback on timing / decision making - The explanations along the way - Good summary of acute stroke processes - Understanding priorities and how critical time management is - Importance of information collection in an efficient and timely manner, effective communication is key to reducing time, while ensuring depth of detail - Take home point for me = don’t waste time, keep moving and intervening - Image interpretation and prioritising information gathering - Highlighting useful elements of history taking and reminder of crucial information, reminder about time pressure, summary of imaging useful - Tips on how to save time during workup of stroke patients - Emphasis on workflow / avoiding preventable delays - Reassurance that I'm adequately assessing patients with acute stroke - Focus on urgency and speed |
| What elements of the TACTICS VR Training module do you feel could be improved upon? | Free-text responses:   - Better Scripting / recordings of clinicians - its good, it was real life, but perhaps a bit too much stuttering, stumbling - Further detail added to virtual environment to mirror real world - Image of CT difficult to see / uncomfortable. Ability to have information repeated or repeat instructions. - No option to pause or go back a step - Critical issues with regard to informed consent were brushed over. Notoriously pre-hospital information is not reliable and information probing for more detail unavailable - Prompting of what to do next to make it more time effective - Once familiar with VR training - repeated practice would be beneficial - Difficult to attain best results when unfamiliar with the technology - Couldn't find the ambulance officer initially, unfamiliar with idea of looking around. Ordinarily, ambulance officer would give a hardcover, and be first person I would interact with - A practice module prior to starting would be helpful. - Doing practice run - Slightly dizzying experience - A bit more information regarding choices made - Would like more information about the errors that were made - The feedback at the end is not specific enough, doesn’t tell me how to improve - Time. Takes a long time for a thing that could take 10 minutes. Too much talking. The waiting room was located next to the bed area. Early days and this technology will grow quickly - Logical errors in coding: despite no time penalties and no sub-optimal decisions the outcome stated the patient was "not processed optimally". The videos which come between the decision points are quite long. That being said I think the information in the videos is very helpful. I think the small piece of additional history from the wife that he 'fell' (during breakfast) should be removed. It is clear the patient has a stroke. The history of a fall creates the potential of head trauma and potential contraindication to thrombolysis. You are asked shortly after this to provide a decision of suitability for thrombolysis/ ECR and it is possible he would be excluded if he had head trauma. If this piece of history was removed from the package then this would be clearer for the purposes of this training package. Otherwise, think it's an excellent package in a format that works for delivering this type of education! - The trainer has to speak more slowly. Flow in ED collateral history needs tidying. - Variety of cases to emphasise different problems (e.g contraindications to thrombolysis, BP control) - Scenario variation - Different paths for different roles (e.g when the CT interpretation is happening, maybe focus on nursing assessment and monitoring during the scan rather than interpreting the CT) - Additional ideas from nursing and allied health - could be expanded with additional info in how to complete an NIHSS and identifying stroke symptoms - separate sessions - As a radiographer I feel like most aspects were not applicable to me - It was more aimed at the MOs role in stroke care instead of nurses (e.g interpreting CTs) - This training was more about the treating team and management of the patient which the radiographer only has a small role in. I was unfamiliar with a lot of the terms used, e.g. stroke scales and treatment acronyms - Incorporate telestroke call as big part of process for us, more detail re obtaining consent, findings on CT a bit unclear - I made a guess based on clinical presentation - Would like to do examination. Not context specific - no opportunity to consult/ telehealth consult stroke physician. - Not all workflows are correct for our site as a telestroke site (i.e a CTP/CTA is not immediately done until the NCCT is reviewed for ICH or established stroke) - Process for telestroke sites are slightly different - The department/ hospitals workings could be a bit more site/ hospital-specific (e.g lack of dedicated stroke team in some hospitals) - Accurately performing NIHSS as often that is the area I feel where I could make up time |
| I am confident in my understanding of workflow practices for acute stroke assessment and treatment | Strongly agree  Agree  Undecided  Disagree  Strongly disagree  (Mean = 4.19 +/- 0.65; Strongly agree = 5) |
| I am confident in my ability to make improvements to how acute stroke care is provided to patients presenting to this hospital | Strongly agree  Agree  Undecided  Disagree  Strongly disagree  (Mean = 4.13 +/- 0.62; Strongly agree = 5) |
| Did the VR module lead to plans to improve acute stroke care? | Yes – Plan to make changes (31)  Yes – Already making changes (8)  No (13) |
| Did you feel motion sickness or nauseous whilst participating in the VR training module | Yes – Could not complete training (4)  Yes – Was able to compete training (6)  No (45) |

VR = virtual reality; Data presented as mean +/- SD.
